# Predicting postoperative urinary retention after lower limb arthroplasty

**DOI:** 10.1308/003588412X13171221591691

**Published:** 2012-07

**Authors:** WKM Kieffer, TPC Kane

**Affiliations:** Portsmouth Hospitals NHS Trust,UK

**Keywords:** Arthroplasty, Urinary retention, Orthopedic surgery, Urology, Postoperative period

## Abstract

A simple scoring system that enables surgeons to make an estimation of the likelihood of postoperative urinary retention (POUR) in patients undergoing lower limb total joint replacement would be a useful one. This would enable selection of high risk patients who merit pre-operative catheterisation in a clean theatre environment rather than risking urinary retention and its associated complications late at night on the ward by junior, inexperienced staff.

The International Prostate Symptom Score (IPSS) is such a scoring system and we assessed its reliability in predicting those male patients likely to go into POUR. We selected all male patients undergoing lower limb total joint arthroplasty under spinal anaesthesia and calculated their IPSS. We found a statistically significant increase in the likelihood of POUR as IPSS rises (*p*=0.0002). We concluded that the IPSS is a quick and easy method of predicting those at risk of POUR, allowing them to be catheterised prophylactically, preventing possible complications.

A recognised complication of surgery and anaesthesia is acute urinary retention with the risk increased in anorectal surgery, hernia repair and orthopaedic surgery as well as with increasing age^1^ and male gender.^2^ Reported rates after lower limb total joint arthroplasty are quoted as between 10.7% and 77.8%.^3,4^

The consequences of acute urinary retention can be severe with potential local and distant sequelae. As well as causing unnecessary pain and discomfort for patients, acute urinary retention can lead to bladder dysfunction and urinary tract infection (UTI) or bacteraemia, thereby acting as a source of infection for any implanted prosthesis.^5^

As junior doctors’ practical procedural experience diminishes in urological emergencies,^6^ the possibility of patients being left with an inexperienced doctor being asked to catheterise the patient in retention rises. A useful tool would be to identify those patients who are at an increased risk of post-operative urinary retention (POUR) so that they might be offered pre-operative catheterisation to avoid possible consequences.

The International Prostate Symptom Score (IPSS) has been suggested as a suitable tool but was found not to be helpful by Sarasin *et al*^7^ while Elkhodair *et al* found it a useful tool for predicting POUR for all male patients undergoing lower limb arthroplasty.^8^ The IPSS is a validated scoring system devised by the American Urological Association consisting of seven questions related to male prostatic symptoms: incomplete emptying, frequency, intermittency, urgency, weak stream, straining and nocturia.^9^ Each item is scored from 1 to 5 on a scale of frequency (or average number of episodes per night in the case of nocturia). It has been found to be reliable regardless of whether it is self-administered or administered by a health professional.^10^

Evidence suggests that the rate of POUR in spinal anaesthesia is higher in foot and ankle surgery^11^ with addition of intrathecal opiates also causing higher rates of urinary retention.^12^ However, the levels of breakthrough analgesia required post-operatively is found to be superior in spinal compared with general anaesthesia,^13^ with intrathecal morphine added providing better analgesia than local anaesthesia alone.^14^

In our department patients undergoing lower limb joint arthroplasty are offered spinal anaesthesia unless contraindicated or declined by the patient given the recognised post-operative benefits of this approach.^15,16^ We aimed to establish the correlation between the IPSS and the rate of POUR in male patients receiving spinal anaesthesia and whether the inclusion of diamorphine increases the rate of POUR.

## Methods

All male patients undergoing lower limb primary total joint arthroplasty between September and November 2010 at Queen Alexandra Hospital, Portsmouth, were included in the study. One hundred consecutive patients had data collected prospectively following consent and IPSS administration. The patients were scored either at their pre-assessment clinic appointment or on the day of surgery, with each score taking less than five minutes to obtain. Patients requiring catheterisation pre-operatively were excluded from the study, as were patients undergoing unicompartmental joint replacement or revision surgery. The null hypothesis was that change in IPSS would have no bearing on the rate of POUR after spinal anaesthesia for lower limb total joint arthroplasty.

Data collected included age, surgical procedure, spinal anaesthetic volumes and concentrations, peripheral nerve blocks and post-operative catheterisation. Post-operatively, ward nursing staff were responsible for observing the patients and, if acute urinary retention was suspected (painful anuria, distended bladder and large residual volume on bladder ultrasonography), the patient was catheterised according to the hospital protocol. The patients had their catheters removed once mobility was resumed.

The contents of the spinal anaesthetic were not controlled due to the number of anaesthetists and preference thereof. Previous history of urinary catheterisation was not routinely recorded.

## Results

Overall, 100 patients were recruited with a median age of 68 years (range: 25–86 years). Of these, 55 patients (55%) underwent total knee arthroplasty compared with 45 (45%) undergoing total hip arthroplasty. These procedures were performed by 15 different consultant surgeons. The median IPSS was 5 (range: 0–34). All patients received intravenous opiates/opioids post-operatively unless contraindicated, eg by allergy.

Eight patients were catheterised prophylactically due to surgeon preference and were therefore excluded from the analysis. Six of these (75%) were catheterised as they had previous POUR. Of the remaining 92 patients, 41.3% (*n*=38) were catheterised due to POUR while 58.7% (*n*=54) were not. All patients had a urinary residual volume of >500ml post-catheterisation, confirming the diagnosis of acute urinary retention.

Almost two-thirds of the patients (63%) had diamorphine included in their spinal anaesthesia. Over a third of the patients (38%) also received peripheral nerve blocks as well as spinal anaesthesia, the majority being femoral nerve blocks.

Dividing patients into mildly symptomatic (IPSS 0–7), moderately symptomatic (IPSS 8–19) and severely symptomatic (IPSS 20–35) gave the following percentages: mild 61.4% (*n*=59), moderate 29.3% (*n*=27) and severe 6.5% (*n*=6). Over a quarter (27.1%) of mildly symptomatic patients went into POUR while the majority of moderately and severely symptomatic patients went into POUR (63.0% and 83.3% respectively). Overall, those patients who went into POUR had a median IPSS of 11 while those who did not had a median score of 4. A chi-squared test for trend showed that there was a statistically significant association between increased IPSS and rate of POUR at the 5% level (*p*=0.0002).

**Table 1:** International Prostate Symptom Score versus percentage of patients requiring urinary catheterisation

IPSS	Number of patients	POUR and catheterisation
Mild (0–7)	59 (61.4%)	27.1%
Moderate (8–19)	27 (29.3%)	63.0%
Severe (20–35)	6 (6.5%)	83.3%

IPSS = International Prostate Symptom Score; POUR = post-operative urinary retention

## Discussion

POUR remains a common cause of post-operative morbidity and discomfort for patients undergoing lower limb total joint arthroplasty. Several other factors have been indicated for giving an increased incidence of POUR such as increased prostate specific antigen, previous history of POUR^17^ and a peak urinary flow of <17ml/s.^18^ Other factors or situations tend to necessitate catheterisation, eg extensive revision surgery and bilateral joint replacements. No consensus, however, exists on a reliable, simple test to use that does not necessitate laboratory tests or urodynamics. As such, a scoring system that could predict those patients at risk of POUR that is easy to use and reliable would be a vital tool for predicting those patients most at risk.

The IPSS is a simple and easy to use system of evaluating male prostatic symptoms. It was developed and validated by the American Urological Association^11^ and is consistent regardless of the questioner.^12^ The IPSS is based on 7 categories, each with a score from 0 to 5: incomplete emptying, frequency, intermittency, urgency, weak stream, straining and nocturia. The scoring system subdivides people into mildly (IPSS 0–7), moderately (8–19) and severely symptomatic (20–35), and can be obtained easily during a pre-assessment visit as was the case in the majority of our patients.

Our findings show that not only was the incidence of POUR increased with a rise in IPSS but that inclusion of morphine in a spinal anaesthetic also increased the rate of POUR. While the rate of POUR does increase with addition of morphine to the spinal anaesthetic,^14^ the analgesic properties do appear much improved with its addition^16^ as they do with the advent of patient controlled analgesia although this also increases the rate of POUR.^19^ Evidence presented by Raffaelli *et al* would appear to show that this side effect among others is not dose dependent, being patient dependent instead.^20^

Limitations of this study include its untested ability to predict POUR in general anaesthesia and the relatively small size of the sample. Furthermore, the differing contents of the spinal anaesthesia were not controlled in this case, as stated, and this was due to anaesthetist preference.

## Conclusions

We feel that the IPSS provides a simple and easy to use scoring system for pre-operative assessment of those patients at high risk of POUR, especially when combined with anaesthetic practices that commonly use spinal anaesthetic with intrathecal morphine. Possible approaches to those patients in the moderately or severely symptomatic groups include pre-operative catheterisation to prevent the likely sequelae from occurring or assessment by a member of the urology services to consider treatment with either α-blockers or 5α-reductase inhibitors, the latter being the option that we would recommend for those screened as severely symptomatic at a minimum.

While we accept that urinary catheterisation is also a risk factor for UTI in orthopaedic procedures,^21^ we would suggest that the risk of UTI following catheterisation away from the confines of the clean conditions of an operating theatre complex with prophylactic administration of antibiotics (for arthroplasty) either recently administered or soon to follow is less than that on the open ward overnight when the qualifications of the staff^8^ and also the aseptic conditions may be lower than desired.

In the case of this study, 22 patients could have been prevented from suffering POUR and its possible sequelae, which, when scaled up to a busy orthopaedic arthroplasty unit, could lead to significant reduction in morbidity related to urinary retention after lower limb total joint arthroplasty and spinal anaesthesia.
